# GPR12 Inhibits Apoptosis in Epithelial Ovarian Cancer *via* the Activation of ERK1/2 Signaling

**DOI:** 10.3389/fonc.2022.932689

**Published:** 2022-07-12

**Authors:** Lu Wang, Da Yang, Yao Zhang, Yisheng Jiao

**Affiliations:** ^1^ Department of General Medicine, Liaoning Cancer Hospital of China Medical University, Liaoning Cancer Hospital & Institute, Shenyang, China; ^2^ Department of Urology, Shengjing Hospital of China Medical University, Shenyang, China; ^3^ Department of Gynecology and Obstetrics, Shengjing Hospital of China Medical University, Shenyang, China

**Keywords:** GPR12, EOC (epithelial ovarian cancer), ERK1/2 signaling, proliferation, apoptosis

## Abstract

Epithelial ovarian cancer (EOC) is one of the most lethal gynecological malignancies in women worldwide. G protein–coupled receptor 12 (GPR12) is a member of G protein–coupled receptors (GPCRs) and plays an important role in the regulation of cell proliferation and survival. However, its role in EOC is underappreciated. In this study, we found that GPR12 is highly expressed in the EOC tissues and can be an ideal biomarker to predict the prognosis of patients with EOC. GPR12 knockdown obviously inhibits the proliferation of EOC cells by inducing cellular apoptosis *in vitro* and *in vivo*. Meanwhile, bioinformatic analysis showed that the inhibitory effect of GPR12 knockdown on the cell viability is closely related with Extracellular signal-regulated kinases 1/2 (ERK1/2) pathway, which has been confirmed by the fact that the activity of ERK1/2 pathway has been significantly blocked in the GPR12 knockdown cells. LM22B-10, ERK1/2 pathway activator, could reverse the inhibited proliferation caused by GPR12 knockdown in the EOC cells. Our findings suggest that GPR12 is involved in the EOC process and is a potential therapeutic target for EOC.

## Introduction

Epithelial ovarian cancer (EOC), including serous carcinomas, mucinous carcinomas, endometrioid carcinomas, and clear cell carcinomas, is the most lethal gynecologic malignancy and accounts for 90% of all ovarian cancer, which is the most common cause of gynecologic cancer death in women. Most patients with EOC are diagnosed at an advanced stage due to the asymptomatic characteristics of EOC (1). Despite the advances in treatments against EOC, such as neoadjuvant chemotherapy and surgical cytoreduction, it still has low 5-year survival rates in recent decades. Therefore, it is of great urgency to search for potential therapeutic targets to improve the prognosis of patients with EOC.

G protein–coupled receptors (GPCRs) constitute the largest superfamily of proteins involved in many crucial physiological and pathological processes, including tumor growth, invasion, survival, and metastasis (2, 3), due to which GPCRs serve as increasingly attractive therapeutic targets. Recently, somatic mutations of multiple GPCRs, including adhesion G protein-coupled receptor L3 (LPHN3), apelin receptor (APLNR), adhesion G protein-coupled receptor B3 (BAI3) and glutamate metabotropic receptor 8 (GRM8), have been reported to be implicated in the development of ovarian cancer, identifying novel therapeutic target in ovarian cancer (4).

GPR12 belongs to the orphan GPCR family without confirmed endogenous ligands (5). It has been shown to be constitutively activated and subsequently initiate Cyclic adenosine monophosphate (cAMP) signaling pathway (6–8). GPR12 plays an important role in cell proliferation and survival, and the activation of ERK 1/2 signaling may be involved in GPR12-mediated cell proliferation (9, 10). Previous studies have proved that GPR12, combined with sphingosylphosphorylcholine (SPC), has positively effect on the proliferation of neuronal precursor cells (11). The upregulated GPR12 has been shown to enhance cell proliferation and survival in Human Embryonic Kidney 293 (HEK293) cells and to induce cell differentiation and neurite outgrowth in PC12 cells via the activation of ERK 1/2 signaling (8, 12). GPR12 has been reported to be relevant to cancer metastasis via modulating the viscoelasticity of metastatic cancer cells by influencing phosphorylation and reorganization of keratin 8 filaments (13).

However, the effects of GRP12 on cell proliferation of EOC and the underlying mechanism have not been reported. In the present study, we characterized the expression of GPR12 and its clinical and prognostic relevance in EOC and found that GPR12 may function as a tumor promotor in EOC. Our findings might provide novel insight into the important role of GPR12 in tumorigenesis and inform a potential therapeutic target against EOC.

## Materials and Methods

### Cell Culture

SKOV3 and CAVO3, two ovarian cancer cell lines, were purchased from the Cell Bank of Chinese Academy of Sciences (Shanghai, China) and maintained in RPMI 1640 medium (Invitrogen, USA) supplemented with 10% fetal bovine serum (BI). Normal ovarian cells, IOSE-80 (a gift from China Medical University), were cultured in Dulbecco's Modified Eagle's Medium (DMEM) (Invitrogen, USA) added with 10% BI. All cells were cultured in a humidified environment at 37°C, 5% CO2.

### Plasmid Construct and Transfection

GPR12 overexpression vector was constructed by inserting GPR12 DNA fragment into pSin-EF2-Nanog-Pur plasmid to replace Nanog as previously described (14). Briefly, pSin-EF2-Nanog-Pur was cut by SpeI and EcoRI New England Biolabs (NEB) and become a linearized plasmid, followed by purification with an agarose gel DNA extraction kit (TaKaRa MiniBEST). Human GPR12 cDNA was obtained from Vigene Biosciences (Shandong, China). Then, clone PCR was performed to amplify and form double-strand DNA (Takara R045Q PrimeSTAR Max DNA Polymerase), which was purified by using an agarose gel DNA extraction kit (TaKaRa MiniBEST). The primer sequences used in the reactions for clone PCR were listed in **Supplementary Table 1**. The 5′ terminals of this set of primers contained 16-nt sequence overlapping with the two terminals of the linearized pSin-EF2-Pur plasmid, respectively, and 5-nt sequence of corresponding restriction enzyme. Then, the DNA fragment was inserted into the linearized plasmid by Gibson Assembly (ClonExpress Ultra One Step Cloning Kit Vazyme C115-02). The empty vector pSin-EF2-Pur (a gift from Zhongshan medical university) was used as a control. The plasmids were verified by sequencing. The short hairpin RNA (shRNA) for human GPR12 was cloned into a hU6-MCS-CBh-gcGFP-IRES-puromycin lentiviral vector (GV493, Genechem, China). The primers used for clone reactions were listed in **Supplementary Table 1**. Cell line stable expression of GPR12, shGPR12#1, and shGPR12#2 was constructed by filtered lentivirus infection using HEK293T cells (a gift from China Medical University) and selected and maintained with puromycin (0.5 mg/ml) for 14 days. The overexpression/knockdown efficiency of GPR12 was evaluated by Western blot.

### Patients and Tumor Tissues

The individual 369 EOC tissues were collected from patients during surgery from the Shengjing Hospital of the China Medical University between January 2011 and December 2019. All patients have been diagnosed on the basis of clinical and pathological evidence, and the tissues were collected and stored at −80°C. Patient consent and approval from the Institute Research Ethics Committee were obtained prior to the use of these clinical materials for research purposes.

### Cell Viability Assay

To determine the effect of GPR12 on cell viability of SKOV3 and CAVO3, CCK-8 assay (APExBIO, USA) was carried out. Cells were seeded into 96-well plates at the density of 5 × 103 cells per well, and the performance was followed to the protocol. Optical density values were read at 450 nm by a microplate reader (Bio-Rad, USA).

### Western Blotting Analysis

Protein extraction was performed by using the radioimmunoprecipitation assay buffer supplemented with phosphatase inhibitor cocktails. Equal volumes of protein lysates were separated using 10% (vol/vol) sodium dodecyl sulfate-polyacrylamide gel electrophoresis (SDS-PAGE) and transferred onto polyvinylidene fluoride (PVDF) membranes (Millipore, Billerica, MA, USA). For immunoblotting, antibodies against G protein-coupled receptors 12 (GPR12) (Abcam, USA), poly (ADP-ribose) polymerase (PARP), caspase-3, Cleaved caspase-3, B cell lymphoma-2 (BCL2), BCL-2-Associated X Protein (BAX), phosphorylation of extracellular-regulated protein kinases 1/2 (p-ERK1/2), total extracellular-regulated protein kinases 1/2 (t-ERK1/2) (Cell Signaling Technology Cambridge, MA, USA), α-tubulin, and β-actin (ProteinTech, China) were used.

### Immunohistochemistry

Immunohistochemistry (IHC) staining was carried out with anti-GPR12 antibody according to the standard protocol. Two independent investigators evaluated the scores of the proportion of tumor cells according to the following criteria: 0 (no positive cells), 1 (positive cells, <10%), 2 (positive cells, 10%–35%), 3 (positive cells, 35%–70%), and 4 (positive cells, >70%). The SI represents staining intensity. Revised version: The staining intensity (SI) was graded as follows: 0 (no staining), 1 (light yellow, faint staining), 2 (yellow brown, moderate staining), and 3 (brown, strong staining). The final immunohistochemical score staining index was the product of the score of SI and the score of the proportion of positive cells (range from 0 to 12).

### Flow Cytometric Analysis

Cell apoptosis was determined with a commercial Annexin V–Fluorescein isothiocyanate (FITC) Apoptosis Detection Kit (Beyorime, China). Briefly, cells were harvested with trypsin, and 1 × 106 cells were resuspended in 100 μl of the binding buffer with 5 μl of Annexin V–FITC and 5 µl PI. Subsequently the cells were incubated at room temperature for 15 min in the dark and then were assayed by a FACScan flow cytometer with CellQuest Pro software.

### Tumor Xenografts

Female 6-week-old BALB/c-nu mice were randomly assigned to two groups (six mice per group). SKOV3 cells (1 × 106) were subcutaneously implanted into the inguinal folds of each nude mouse. Tumor height and width were measured with a caliper every 2 days to calculate tumor volume (= width2 × height × π/6). For subcutaneous tumor growth, the maximum single tumor cannot exceed 1.2 cm in diameter in mice, and no experiments in this study generated tumor burden over this limit. Tumor tissues were collected from the nude mice, fixed in 4% paraformaldehyde, and embedded in paraffin.

### Statistical Analysis

All the data were analyzed using GraphPad 5.0 software (USA). The results are presented as means ± standard deviation (SD). Student’s t-test was used to was used to make comparisons between two groups. One-way ANOVA was performed to make comparisons among multiple testing. Chi-square test was applied to detect the relationship between GPR12 expression and clinicopathological characteristics. Kaplan–Meier survival curves were analyzed by log-rank test. Multivariable cox regression analysis was used to determine independent risk factors of EOC. A value of P < 0.05 was considered statistically significant.

## Results

### GPR12 Is Upregulated in Epithelial Ovarian Cancer and Predicts Poor Prognosis

**Figure 1:**
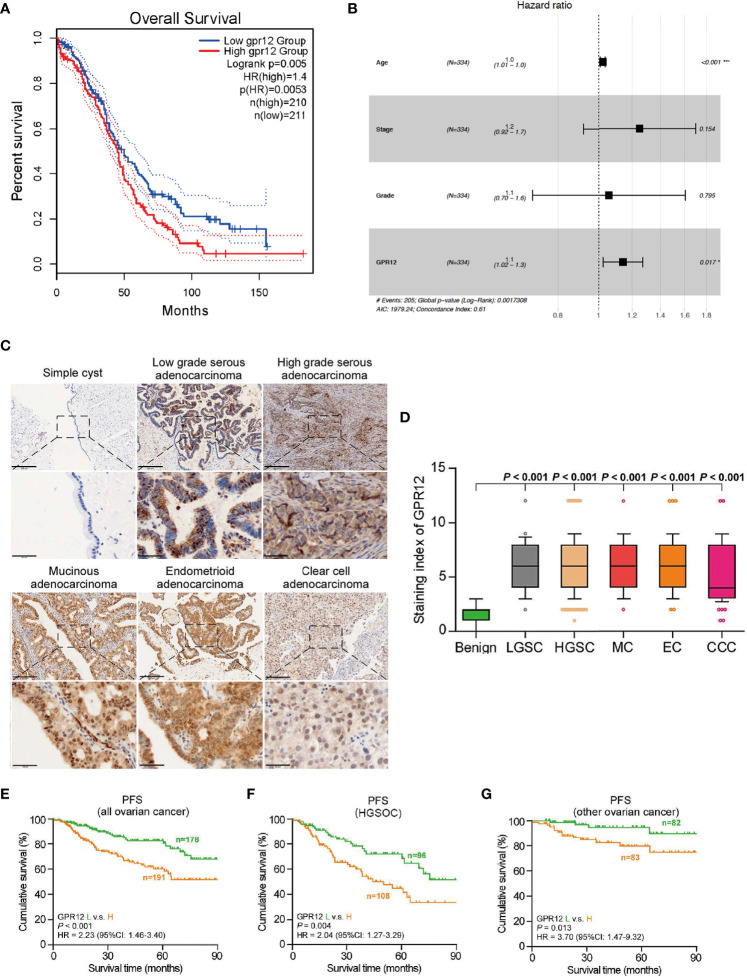
GPR12 is overexpressed in the EOC and predicts the prognosis of patients with EOC. **(A)** Association of GPR12 expression with overall survival (OS) was evaluated using Kaplan–Meier analysis in patients in the TCGA EOC database. **(B)** Multivariate Cox analysis of GPR12 and clinical phenotype characters in TCGA-OV database. **(C)** GPR12 expression in clinical samples was stained by immunohistochemistry (IHC). Scale bar = 200 μm in the upper panels and 50 μm in the below panels. **(D)** GPR12 expression was significant higher in different histological types of EOC. **(E–G)** Association of GPR12 expression with progression-free survival (PFS) was evaluated using Kaplan–Meier analysis on 369 patients with EOC.

To investigate whether the prognosis of EOC is associated with GPR12 expression, we first performed Kaplan–Meier survival analysis through Gene Expression Profiling Interactive Analysis (GEPIA, http://gepia.cancer-pku.cn/) by using transcriptome and survival data from The Cancer Genome Atlas (TCGA) dataset. Results showed a significant reduction in overall survival (OS) rate in patients with EOC with high GPR12 mRNA expression than those with low GPR12 mRNA expression (**Figure 1A**). In addition, we also performed a Cox regression analysis by using EOC data from TCGA database. GPR12 mRNA expression and clinical characteristics including age, stage, and grade were selected into the multivariable Cox regression model. Results showed that GPR12 expression (p = 0.017, hazard ratio = 1.1), and age (p < 0.01, HR = 1) were significantly associated with EOC prognosis (**Figure 1B**). Then, a total of 369 patients with EOC were recruited in our study. The GPR12 protein expressions in low-grade serous ovarian cancer (LGSOC), high-grade serous ovarian cancer (HGSOC), mucinous adenocarcinoma (MC), endometrioid adenocarcinoma (EC), clear cell adenocarcinoma (CCA), and normal ovarian epithelial tissues were examined by immunohistochemical staining, respectively. Significantly increased GPR12 expression was observed in all histological subtypes of EOC tissues compared with the normal ovarian epithelial tissues (**Figures 1C, D**). All patients with EOC composed of HGSOC patients and other patients with EOC, were further divided into GPR12 low expression group and GPR12 high expression group based on the immunohistochemical results, and clinicopathological characteristics of patients were assessed, respectively. As shown in **Table 1**, the patients with high GPR12 expression have higher proportion of the positive in progression status than the patients with low GPR12 expression. In addition, the patients with high GPR12 expression have lower sensitivity to first chemotherapy than the patients with low GPR12 expression. Furthermore, progression-free survival (PFS) of all patients with EOC, patients with HGSOC, and the patients with other types of EOC was assessed, separately. Our results showed that PFS was significantly decreased in all patients with EOC, HGSOC, or the other types of EOC with high GPR12 levels than that in the patients with low GPR12 levels, respectively (**Figures 1E–G**). These results suggest that GPR12 may play a critical role in the progression of EOC and serves as a biomarker for prognosis in EOC.

**Table 1:** The relationship between GPR12 protein level and clinical pathological characteristics in 369 patients with epithelia ovarian cancer.

Parameters	Number of Cases	GPR12 IHC-SI		P-values
		Low	High	
Age				
<50	139	72	67	0.287
≥50	230	106	124
Histologic				
Type I	165	82	83	0.614
Type II	204	96	108	
FIGO stage				
I–II	246	120	126	0.786
III–IV	123	58	65	
Progression status (in 3 y)				
Positive	65	19	46	<0.001
Negative	145	81	64	
First chemotherapy response (at 6 m)				
Sensitivity	78	41	37	0.012
Resistance	42	12	30	

(1) Histologic type I included low-grade serous adenocarcinoma, mucinous adenocarcinoma, endometrioid adenocarcinoma, and clear cell adenocarcinoma. Histologic type II included high-grade serous adenocarcinoma and undifferentiated carcinoma. (2) IHC, immunohistochemical staining index; FIGO, The International Federation of Gynecology and Obstetrics.

### GPR12 Regulates EOC Cell Proliferation and Apoptosis *In Vitro*

**Figure 2:**
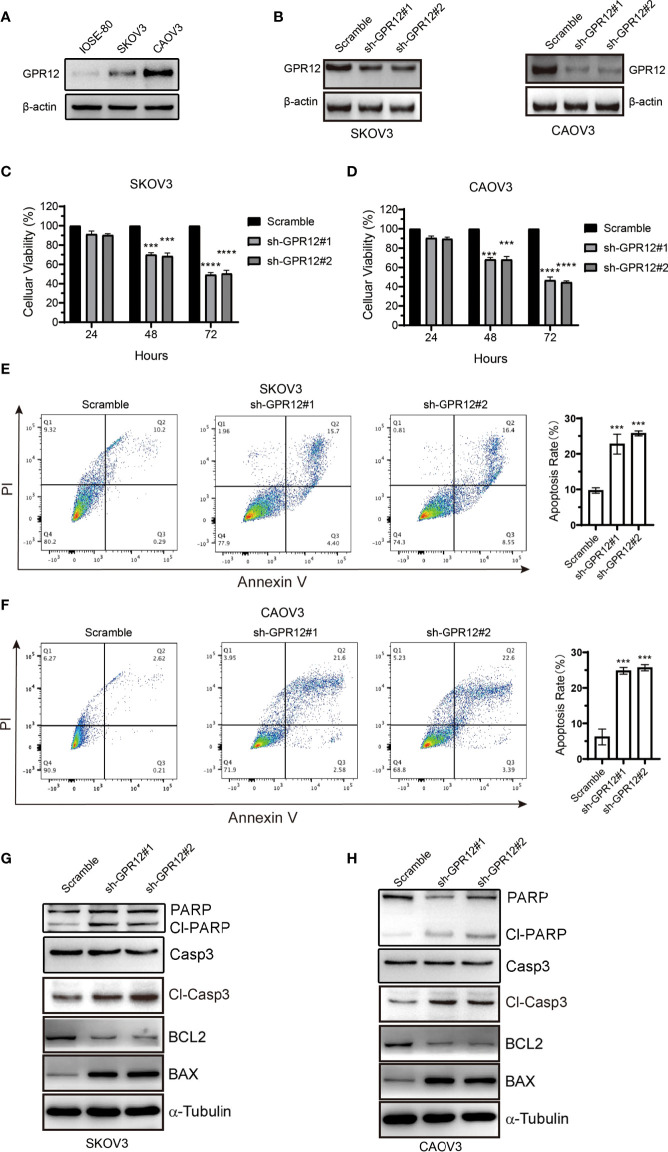
GPR12 knockdown with GPR12-shRNAs inhibits the viability and induces apoptosis of EOC cells. **(A)** Protein expression of GPR12 in normal ovarian epithelial cell (IOSE-80) and two epithelial ovarian cancer cell lines (SKOV3 and CAOV3) was determined by Western blot. **(B)** GPR12 expression was knocked down with GPR12-shRNAs in SKOV3 and CAOV3 cells. **(C, D)**, The cell viabilities of SKOV3 and CAOV3 cells were determined when GPR12 was knocked down at 24, 48, and 72 h, respectively. **(E, F)**, The cellular apoptosis was analyzed with Flow Cytometry using Annexin V^+^ and PI^+^ staining in SKOV3 and CAOV3 cells transfected with/without GPR12-shRNAs. **(G, H)** Western Blot method was used to detect the expressions of PARP, capase3, cleaved caspase3, Bcl-2, and BAX in SKOV3 and CAOV3 cells after GPR12 knockdown.

To investigate the role of GPR12 in EOC, SKOV3, and CAOV3 cell lines was selected for the study. First, we examined the protein level of GPR12 in normal ovarian epithelial cell line (IOSE-80) and two EOC cell lines (SKOV3 and CAOV3) by using Western blot. Results showed that the protein expression of GPR12 was significantly increased in SKOV3 and CAOV3 cells compared with IOSE-80 cells (**Figure 2A**). Two different small hairpin RNA (shRNA) (sh-GPR12-1 and sh-GPR12-2) were used to knock down the expression of GPR12 in SKOV3 and CAOV3 cells (**Figure 2B**). CCK-8 assay was used to determine the cell viabilities of SKOV3 and CAOV3 cells transfected with GPR12 shRNAs or empty vector (scramble). After 24 h, there was no significant change in cell viability of SKOV3 and CAOV3 cells transfected with GPR12 shRNAs than that in the cells transfected with scramble sequences. However, 48 and 72 h after the transfection, GPR12 downregulation significantly decreased cell viability in both SKOV3 and CAOV3 cells compared with the scramble group, respectively (**Figures 2C, D**). Furthermore, we overexpressed GPR12 in SKOV3 and CAOV3 cell lines (**Supplementary Figure 1A**) and performed CCK8 assays to evaluate cell viability. Results showed that GPR12 overexpression promoted the viability and proliferation of SKOV3 and CAOV3 cell lines compared with control group (**Supplementary Figures 1B, C**).

Then, we further assessed the effect of GRP12 on apoptosis of SKOV3 and CAOV3 cells using flow cytometric analysis. Results showed that GPR12 downregulation significantly increased Annexin V–positive cell population in both SKOV3 and CAOV3 cells compared with scramble group, respectively (**Figures 2E, F**), whereas GPR12 overexpression exerted opposite effects in both SKOV3 and CAOV3 cells (**Supplementary Figures 1D, E**). Western blot results showed that GPR12 knockdown significantly increased the protein levels of BAX, cleaved caspase-3, and cleaved PARP and decreased the expression of BCL-2 in both SKOV3 and CAOV3 cells compared with scramble group, respectively. Meanwhile, GPR12 knockdown significantly decreased the protein level of PARP in CAOV3 cells. However, no significant change in caspase3 protein level was observed in both SKOV3 and CAOV3 cells compared with scramble group (**Figures 2G, H**). These results suggested that GPR12 knockdown inhibits EOC cell proliferation by inducing cellular apoptosis. In GPR12-overexpressed SKOV3 and CAOV3 cells, significantly decreased protein levels of cleaved PARP and cleaved caspase-3 and significantly increased BCL-2 protein expression were observed (**Supplementary Figure 1F**). Although there was no change in BAX protein level in SKOV3 and CAOV3 cells compared with control group, the protein expression ratio of BCL-2 to BAX, a critical index for evaluating endogenous cell apoptosis (15), was significantly increased in SKOV3 and CAOV3 cells. In addition, no significant change in the protein levels of PARP and caspase3 was observed in both SKOV3 and CAOV3 cells compared with control group (**Supplementary Figure 1F**). These results suggest that GPR12 overexpression could inhibit the apoptosis of SKOV3 and CAOV3 cells.

### ERK1/2 Pathway Is Involved in EOC Cell Apoptosis Regulated by GPR12

**Figure 3:**
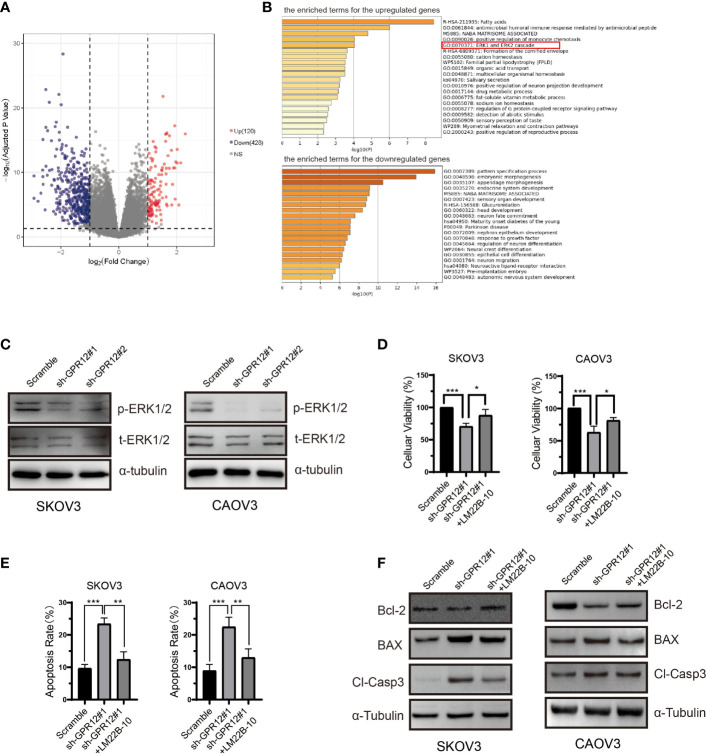
ERK1/2 pathway is involved in the proliferation inhibition induced by GPR12 knockdown in EOC cells. **(A)** Volcano plot of the upregulated and downregulated DEGs. **(B)** The enriched terms for the upregulated and downregulated DEGs. **(C)** Expressions of p-ERK1/2 and t-ERK1/2 in SKOV3 and CAOV3 cells transfected with GPR12-shRNAs were determined with Western blot. **(D)** Effects of ERK1/2 activator LM22B-10 on the viability of SKOV3 and CAOV3 cells transfected with/without GPR12-shRNAs. **(E)** Effects of ERK1/2 activator LM22B-10 on the apoptosis of SKOV3 and CAOV3 cells transfected with/without GPR12-shRNAs. **(F)** The protein expressions of Bcl-2, BAX, and cleaved caspase 3 in SKOV3 and CAOV3 cells after GPR12 knockdown with/without the presence of LM22B-10.

To investigate the potential underlying mechanisms by which GPR12 knockdown enhanced EOC cell apoptosis, bioinformatic analysis was carried out to explore and reveal the possible molecular mechanism. First, differential expressed genes (DEGs) between the high GPR12 expression and the low GPR12 expression were screened using the R software with the “DEGSeq2” package. There were 548 DEGs, including 428 downregulated and 120 upregulated genes (adjusted P < 0.05 and |logFC| ≥ 1), as shown in **Figure 3A**. Then, gene ontology (GO) and pathway enrichment analysis was conducted using Metascape online tool to explore the functional characteristics of the DEGs. The analysis results showed that upregulated DEGs were significantly enriched in “fatty acids”, “antimicrobial humoral immune response mediated by antimicrobial peptide”, “NABA MATRISOME ASSOCIATED”, “positive regulation of monocyte chemotaxis”, and “ERK1 and ERK2 cascade” (**Figure 3B**). For downregulated DEGs, significant enrichments were identified in “pattern specification process”, “embryonic morphogenesis”, “appendage morphogenesis”, “endocrine system development”, and “NABA MATRISOME ASSOCIATED” (**Figure 3B**).

On the basis of the computing method of enrichment analysis, changes in expression level of key genes contributing to enrichment in the gene set of ERK1 and ERK2 cascade may be the protein kinase cascade or downstream genes. We further performed correlation analysis of GPR12 mRNA level with ERK1/2 cascade expression including MAP3K1, MAP2K1, MAP2K2, and MAPK1 by using ovarian cancer data from TCGA database. Results showed that no significant correlation was observed between GPR12 level and ERK1/2 cascade expression (**Supplementary Figure 3A**). Accumulating research studies have shown that the phosphorylation of ERK1/2 is a key effector to exert an influence on cell proliferation and death by translocating into nucleus (16). More importantly, GPR12 has been reported to promote HEK 293T proliferation and induce neurite outgrowth in PC12 cells via the activation of ERK1/2 (8, 12). Therefore, to investigate whether the EOC cell apoptosis induced by GPR12 knockdown was mediated by ERK1/2 pathway, we measured the level of pERK1/2 by using Western blot. Results showed that GPR12 knockdown significantly reduced the phosphorylation levels of ERK1/2 in both SKOV3 and CAOV3 cells compared with scramble group (**Figure 3C**), whereas ERK1/2 phosphorylation was significantly increased in the GPR12-overexpressed SKOV3 and CAOV3 cells (**Supplementary Figure 1G**). To further investigate the role of ERK1/2 pathway in EOC cell apoptosis induced by GPR12 knockdown, both SKOV3 and CAOV3 cells were pretreated with LM22B-10, the selective activator of ERK1/2. As shown in **Figure 3D**, the decreased cellular viability induced by GPR12 knockdown was significantly attenuated by pretreatment with LM22B-10 in both SKOV3 and CAOV3 cells. In addition, the enhanced cell apoptosis induced by GPR12 knockdown was significantly inhibited by pre-incubation with LM22B-10 in both SKOV3 and CAOV3 cell lines (**Figure 3E**). Western blot results also proved that LM22B-10 can rescue the upregulated expressions of BAX and the cleaved caspase 3 in the cells of GPR12 knockdown (**Figure 3F**).

All these results proved that ERK1/2 pathway is involved in EOC cell apoptosis regulated by GPR12.

### Knockdown of GPR12 Inhibits EOC Proliferation and Promotes Apoptosis *In Vivo*

**Figure 4:**
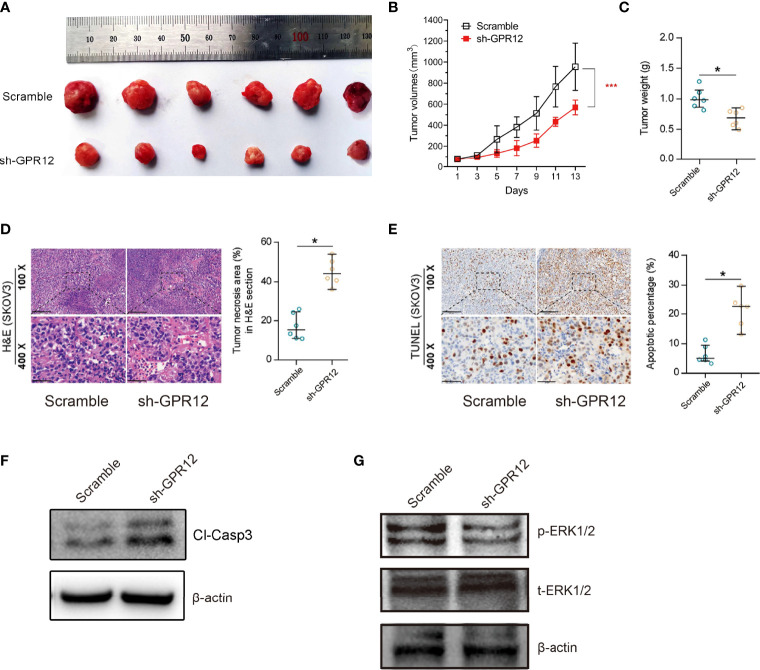
GPR12 knockdown inhibits tumor growth of EOC cells *in vivo*. **(A)** Representative images of the SKOV3 cancer cell tumor xenograft from each mouse (n = 6 mice per group). **(B)** Tumor growth in xenograft mice was recorded. **(C)** The weight of tumors was measured. **(D)** HE staining on the xenograft tumors. Scale bar = 100 μm in the upper panels and 20 μm in the below panels. **(E)** The apoptosis was analyzed by TUNEL staining. Scale bar = 100 μm in the upper panels and 20 μm in the below panels. **(F)** Protein expression of cleaved caspase3 was determined by Western blot in the tumor tissues from the xenograft mice. **(G)** Protein expression of p-ERK1/2 and t-ERK1/2 was determined in the tumor tissues from the xenograft mice.

To further clarify the effects of GPR12 on tumor progression *in vivo*, a subcutaneous tumor xenograft model in nude mice was established and TUNEL assay was performed to evaluate cancer cell apoptosis and growth *in vivo*. About 5 × 105 SKOV3 cells transfected with GPR12-shRNAs or scramble vector were subcutaneously implanted into the inguinal folds of nude mice. Around 14 days later, tumor volumes and weight were measured and recorded when the average tumor volume reached around 100 mm3. As shown in **Figures 4B, C** knockdown of GRP12 significantly decreased tumor volumes and tumor weight in nude mice compared with those from scramble group. The representative images of individual tumors are shown **Figure 4A**. In addition, Hematoxylin-Eosin (HE) staining and TdT-mediated dUTP-biotin nick end labeling (TUNEL) staining showed that GPR12 knockdown significantly increased the tumor necrotic area (**Figure 4D**) and the number of apoptotic cells in the tumor tissues of mice compared with scramble group, respectively (**Figure 4E**). Western bolt results showed that cleaved caspase3 was significantly increased in tumor lysates of GPR12 knockdown group compared with scramble (**Figure 4F**), which indicated that GPR12 knockdown may lead to the tumor regression of EOC by inducing apoptosis. Analysis of tumor lysates from scramble and GPR12 knockdown mice showed a significant decrease in phosphorylated ERK1/2 protein levels in GPR12 knockdown tumors (**Figure 4G**).

Results of the *in vivo* and *in vitro* studies demonstrate that GPR12 knockdown contributes to the cell apoptosis in EOC cells via ERK1/2 pathway.

## Discussion

As the largest family of cell-surface molecules involved in signal transduction, GPCRs have received considerable attention as therapeutic targets for cancers (3). In the present study, we demonstrated that GPR12 was significantly overexpressed in all four pathological types of EOC tissues and the high expression level of GPR12 is closely related to the poor prognosis within the patients with EOC. Furthermore, our results identified the indispensable roles of GPR12 on maintaining the EOC cells viability through inhibiting apoptosis. Mechanistically, the effect of GPR12 in regulating cell apoptosis by the activation of ERK1/2 was confirmed in SKOV3 and CAVO3 cells.

GPR12 was originally identified in the cDNA library from a rat in 1991 (17), and human GPR12 was ultimately cloned in 1995 and mapped to chromosomal region 13q12 (18). Although a series of studies have verified the physiological and pathological importance of GPR12 during multiple events, such as neurite outgrowth and neuronal development (8), obesity and metabolic disorders (19), oocyte maturation (20), and cell survival and proliferation (12), the role of GPR12 in cancer development remains largely unknown. In pancreatic cancer cells (PANC-1) cells, Park et al. observed that overexpression of GPR12 induced K8 phosphorylation and perinuclear reorganization, promoting migration and invasion by changing the viscoelasticity (13). However, Zhang et al. demonstrated that GPR12 as a potential tumor suppressor–mediated cell migration and apoptosis in esophageal cancer and hypopharyngeal cancer (21), indicating that the characteristics of GPR12 varied in different types of cancer. Here, we found that GPR12 mRNA expression is extremely low in the majority of cancer types and significantly decreased in glioblastoma multiforme (GBM), brain lower grade glioma (LGG), and skin cutaneous melanoma (SKCM) tissues than matched normal tissues through pan-cancer analysis of GEPIA (**Supplementary Figure 2A**). Meanwhile, in addition to EOC, results showed that a significant reduction in disease free survival only in patients with pancreatic adenocarcinoma (PAAD) with low GPR12 mRNA expression than those with high GPR12 mRNA expression (**Supplementary Figure 2B**). Interestingly, the mRNA expression of GPR12 is enhanced in ovarian cancer tissues relative to normal tissues (**Supplementary Figure 2B**), which is consistent with IHC results that GPR12 was upregulated in human EOC tissues. Furthermore, results of survival analysis and multivariable Cox analysis indicate that GPR12 high expression level predicted poor prognosis and could be an independent risk factor in patients with EOC. A recent study has demonstrated that GPR12 is predominantly expressed in the ovary of vertebrates (22). Interestingly, progesterone increased GPR12 mRNA level in cultured ovarian granulosa cells (GCs) in a dose-dependent manner, suggesting that GPR12 expression may be upregulated by gonadal steroid hormones in vertebrates (22). On the basis of the roles of gonadotropins and endogenous hormones in the pathogenesis of EOC (23, 24), GPR12 expression may be increased in patients with EOC. In addition, it has been widely accepted that protein ubiquitination and degradation are closely related to protein levels. We firstly investigated the ubiquitinated ligases associated with GPR12 through the UBiBrowser database (http://ubibrowser.bio-it.cn/) and found that NEDD4, SMURF1, ITCH, and SMURF2 may be the ubiquitination E3 ligases targeting GPR12 for ubiquitin-mediated proteasome degradation (**Supplementary Figure 2C**). Then, we analyzed the mRNA expression of these ubiquitination E3 ligases in different tumor tissues using TCGA dataset. Results showed that the mRNA levels of NEDD4, SMURF1, ITCH, and SMURF2 were decreased in OV tissues compared with normal tissues (**Supplementary Figure 2D**). Given the decreased GPR12 expression in GBM and LGG, as a control, increased mRNA levels of NEDD4, SMURF1, ITCH, and SMURF2 were observed in GBM and LGG tissues. Therefore, we speculated that increased GPR12 protein level in patients with EOC may be attributed to the tissue-specific enhancement of ubiquitination E3 ligases targeting GRP12. However, the exact cause of increased GPR12 expression in patients with EOC needs to be further explored.

Previous studies have shown that GPR12, combined with SPC, enhanced the proliferation of neuronal precursor cells (11). In addition, GPR12 overexpression has been indicated to improve cellular proliferation and survival in HEK293 cells (12). We found that GPR12 knockdown inhibited EOC cell proliferation via inducing cell apoptosis *in vitro* and *in vivo* and that GPR12 overexpression significantly promoted EOC cell viability and inhibited apoptosis *in vitro*. Apoptosis acts a protective mechanism when cells confront injury (25), and defective apoptosis is an indispensable contributing factor in cancer development and progression (26). It has been verified that BAX promotes cell apoptosis through antagonizing the inhibitory effect of Bcl-2 on apoptosis (27, 28). We found that the expression of apoptosis markers BAX was significantly increased and that the BCL-2 expression was downregulated in the GPR12 knockdown cells. As the terminal cleavage enzyme of both the endogenous and exogenous apoptotic pathways (29), the cleaved caspase-3 was obviously increased. Meanwhile, as the substrate of activated caspase3 (30), cleaved PARP was increased. These changes of the apoptosis-related molecules indicated that GPR12 regulates EOC cell proliferation and apoptosis.

Recently, accumulating pieces of evidence have identified several signaling pathways involved in apoptosis of EOC cells, including Janus kinase/signal transducer and activator of transcription 3 (JAK/STAT3), Wnt/β-Catenin, mesenchymal-epithelial transition factor (MET)/hepatocyte growth factor (HGF), MAPK/ERK, and PI3K/AKT/mTOR (31). Therefore, the mRNA data of EOC were downloaded from TCGA database, and the bioinformatic analysis was performed using TCGA dataset to further explore how GPR12 affected apoptosis of EOC cells. The differential expression analysis was performed using “edgeR” package followed by pathway enrichment analysis with Metascape online tool. We found that there were 120 genes upregulated and 428 genes downregulated in the high–GPR12 expression group when compared with the low–GPR12 expression group. In addition, the enriched term analysis for DEGs showed that GPR12 was closely related to many biological processes, including “ERK1 and ERK2 cascade”. This gene set is identified as an intracellular protein kinase cascade containing ERK1 or ERK2 (MAPKs), a MEK (a MAPKK), and a MAP3K and consists of 341 genes including downstream genes of p-ERK1/2. We performed correlation analysis of GPR12 mRNA level with ERK1/2 cascade expression including MAP3K1, MAP2K1, MAP2K2, and MAPK1 and found that there was no significant correlation between GPR12 level and ERK1/2 cascade expression. In addition, kinases in each tier phosphorylate and activate the kinase in the downstream tier to transmit a signal within a cell. Accumulating research studies have shown that the phosphorylation of ERK1/2 plays an important role in cell proliferation and death by translocating into nucleus (16). More importantly, GPR12 has been reported to promote HEK 293T proliferation and induce neurite outgrowth in PC12 cells via the activation of ERK1/2 (8, 12). In this study, Western blot results showed that GPR12 knockdown significantly reduced the phosphorylation of ERK1/2 but did not change the total level of ERK1/2 in both SKOV3 and CAOV3 cells compared with scramble group, suggesting that GPR12 may not change the expression pattern but induce phosphorylation of ERK1/2 to transmit a signal. Conversely, GPR12 overexpression significantly increased the phosphorylation of ERK1/2. In addition, rescue experiments indicated that LM22B-10, the activator of ERK1/2 pathway, could partially reverse the inhibited proliferative and the increased apoptosis rate in SKOV3 and CAOV3 cells transfected with GPR12 shRNAs, verifying the possible mechanism that GPR12 regulates EOC cell proliferation and apoptosis via activation of ERK1/2.

In conclusion, we found that OS and PFS are reduced in patients with EOC with high GPR12 expression. Furthermore, our studies show that GPR12 plays an essential role in EOC proliferation and apoptosis, suggesting its potential as a valuable therapeutic target. Mechanistically, GPR12 could regulate EOC cell proliferation and apoptosis through ERK1/2 signaling pathway. However, further investigation is needed to identify the biological function of GPR12 and determine whether the receptor could be a therapeutic target for EOC.

## Data Availability Statement

Publicly available datasets were analyzed in this study. This data can be found here: https://portal.gdc.cancer.gov/TCGA ovarain cancer datasets.

## Ethics Statement

The studies involving human participants were reviewed and approved by Medical Ethics Committee of Shengjing Hospital Affiliated to China Medical University. The patients/participants provided their written informed consent to participate in this study. The animal study was reviewed and approved by Medical Ethics Committee of Shengjing Hospital Affiliated to China Medical University.

## Author Contributions

LW and DY performed most of the experiments. YJ designed the experiments and analyzed the data. DY wrote the paper. YZ provided clinical tissue samples. All authors read and approved the final manuscript.

## Funding

This study was supported by the National Natural Science Foundation of China (No. 82072887) and Joint Project for Key Research and Development Programme of Liaoning province (No. 2020JH2/10300143)

## Conflict of Interest

The authors declare that the research was conducted in the absence of any commercial or financial relationships that could be construed as a potential conflict of interest.

## Publisher’s Note

All claims expressed in this article are solely those of the authors and do not necessarily represent those of their affiliated organizations, or those of the publisher, the editors and the reviewers. Any product that may be evaluated in this article, or claim that may be made by its manufacturer, is not guaranteed or endorsed by the publisher.

## Supplementary Material

Supplementary Figure 1 GPR12 overexpression promotes the viability and inhibites the apoptosis of EOC cells and the expression of p-ERK1/2 and t-ERK1/2 in EOC cells transfected with GPR12 vector. **(A)**, GPR12 was overexpressed in SKOV3 and CAOV3 cells. **(B, C)**, The cell viabilities of SKOV3 and CAOV3 cells were determined when GPR12 was overexpressed at 24h, 48h and 72h, respectively. **(D, E)**, The cellular apoptosis was analyzed with Flow Cytometry using Annexin V^+^ and PI^+^ staining in SKOV3 and CAOV3 cells transfected with/without GPR12 vector. **(F)**, Western Blot method was used to detect the expressions of PARP, capase3, cleaved caspase3, Bcl-2 and BAX in SKOV3 and CAOV3 cells after GPR12 overexpression. **(G)**, Expressions of p-ERK1/2 and t-ERK1/2 in SKOV3 and CAOV3 cells transfected with GPR12 vector were determined with Western Blot.

Supplementary Figure 2 The mRNA level and survival analysis of GPR12 and mRNA level of E3 ubiquitination ligase targeting GPR12 in ovarian caner and other cancer types. **(A)**, mRNA level of GPR12 in tumor and normal tissues of 33 cancer types. **(B)**, Correlation of GPR12 expression with disease free survival (DFS) was evaluated using Kaplan–Meier analysis in the TCGA pancreatic adenocarcinoma (PAAD) patients. **(C)**, mRNA level of multiple E3 ubiquitination ligases in ovarian cancer (OV), glioblastoma multiforme (GBM) and brain lower grade glioma (LGG) by using TCGA database.

Supplementary Figure 3 Correlation analysis of GPR12 and ERK1/2 cascade in OV data from TCGA database. **(A–D)**, Correlation analysis of GPR12 mRNA level with ERK1/2 cascade expression including MAP3K1 **(A)**, MAP2K1 **(B)**, MAP2K2 **(C)** and MAPK1 **(D)** by analyzing OV data from TCGA database.

Supplementary Table 1 Primers used in the reactions for clone PCR.
