# Supplementary figures and images for: GPR12 Inhibits Apoptosis in Epithelial Ovarian Cancer via the Activation of ERK1/2 Signaling
Source: Front Oncol. 2022 Jul 12;12:932689. doi: 10.3389/fonc.2022.932689 (PMC9316591; doi:10.3389/fonc.2022.932689)

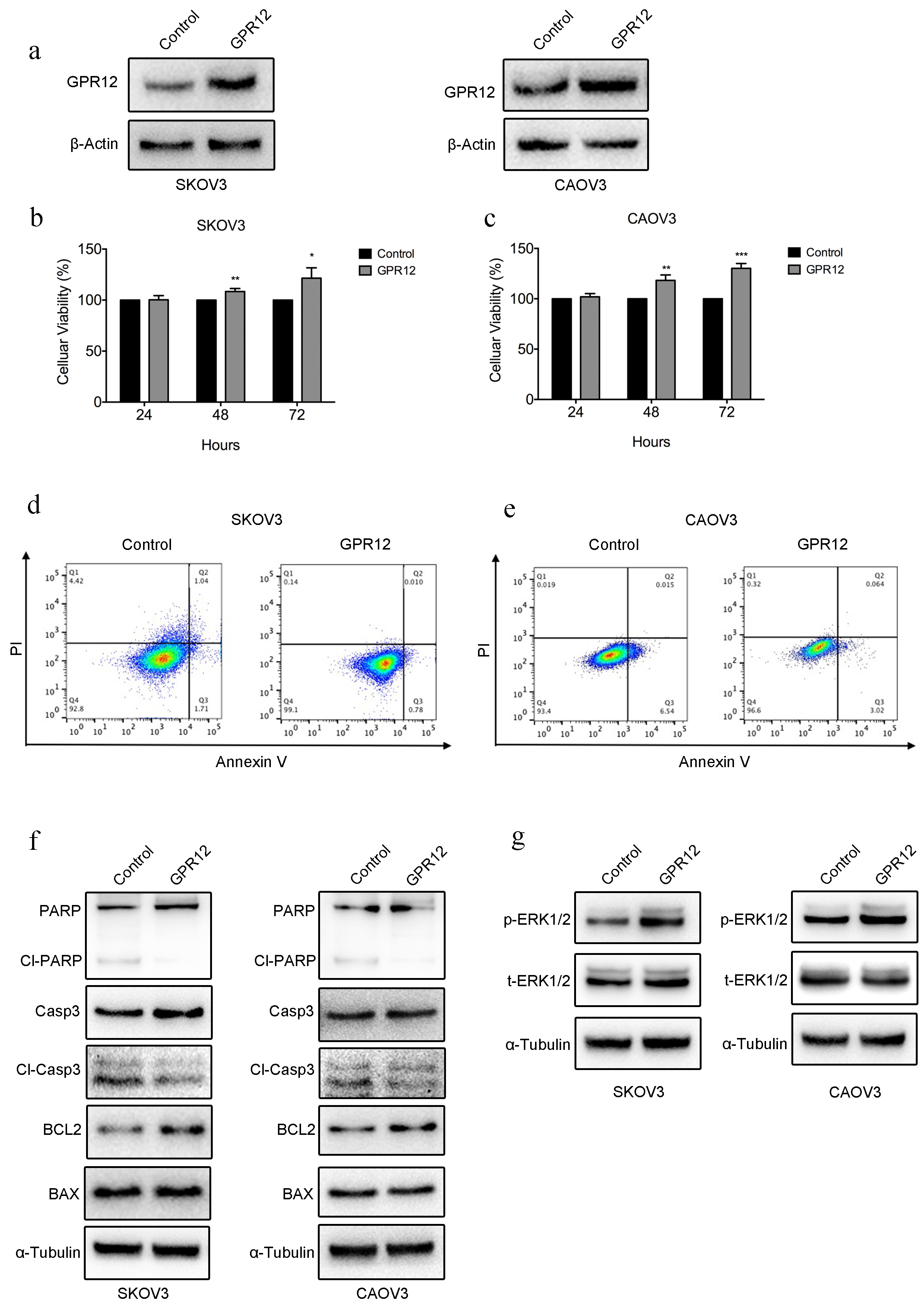

Supplement: Supplementary Figure 1 — GPR12 overexpression promotes the viability and inhibites the apoptosis of EOC cells and the expression of p-ERK1/2 and t-ERK1/2 in EOC cells transfected with GPR12 vector. (A), GPR12 was overexpressed in SKOV3 and CAOV3 cells. (B, C), The cell viabilities of SKOV3 and CAOV3 cells were determined when GPR12 was overexpressed at 24h, 48h and 72h, respectively. (D, E), The cellular apoptosis was analyzed with Flow Cytometry using Annexin V+ and PI+ staining in SKOV3 and CAOV3 cells transfected with/without GPR12 vector. (F), Western Blot method was used to detect the expressions of PARP, capase3, cleaved caspase3, Bcl-2 and BAX in SKOV3 and CAOV3 cells after GPR12 overexpression. (G), Expressions of p-ERK1/2 and t-ERK1/2 in SKOV3 and CAOV3 cells transfected with GPR12 vector were determined with Western Blot. [file Image_1.jpeg]

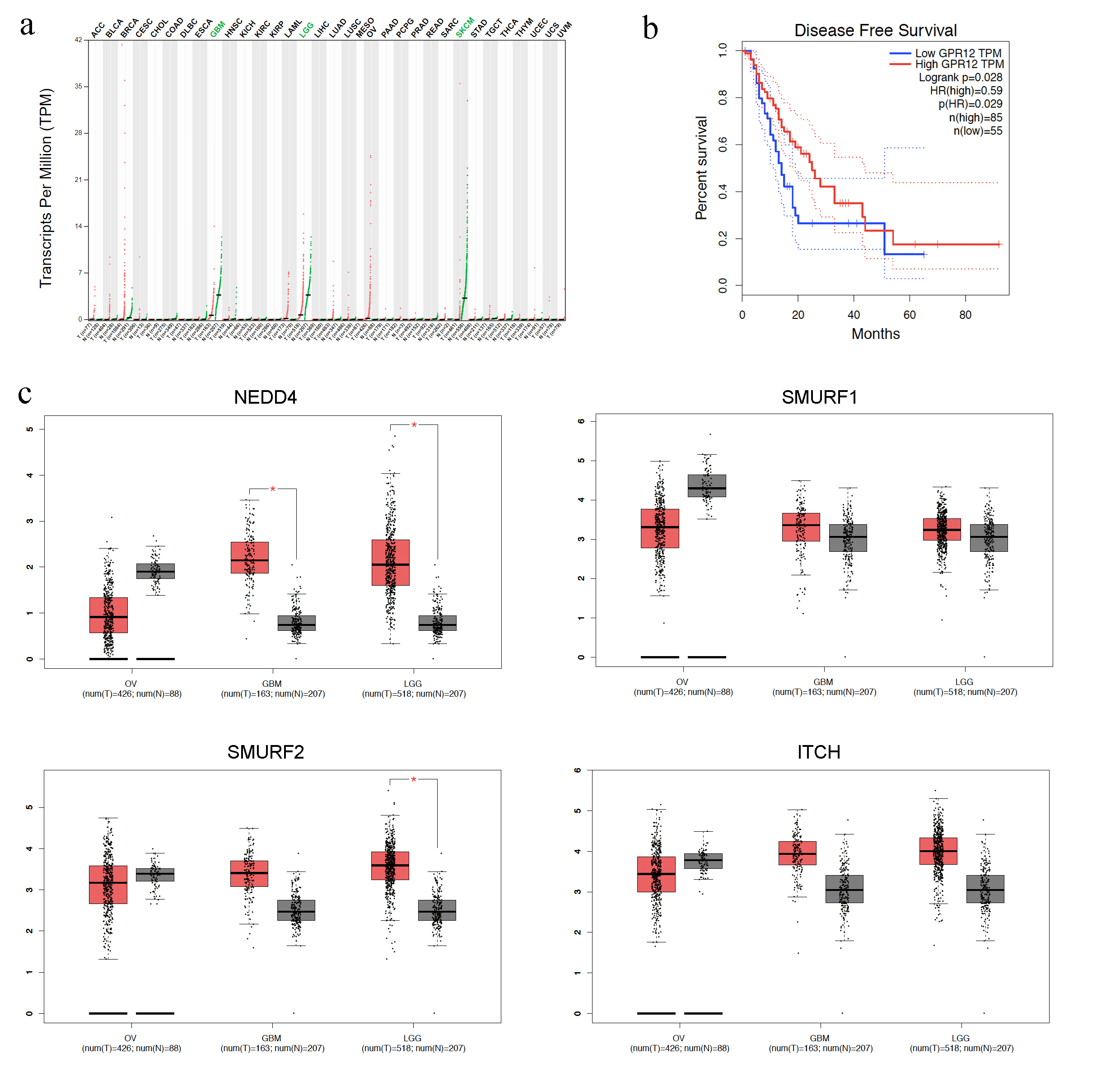

Supplement: Supplementary Figure 2 — The mRNA level and survival analysis of GPR12 and mRNA level of E3 ubiquitination ligase targeting GPR12 in ovarian caner and other cancer types. (A), mRNA level of GPR12 in tumor and normal tissues of 33 cancer types. (B), Correlation of GPR12 expression with disease free survival (DFS) was evaluated using Kaplan–Meier analysis in the TCGA pancreatic adenocarcinoma (PAAD) patients. (C), mRNA level of multiple E3 ubiquitination ligases in ovarian cancer (OV), glioblastoma multiforme (GBM) and brain lower grade glioma (LGG) by using TCGA database. [file Image_2.jpeg]

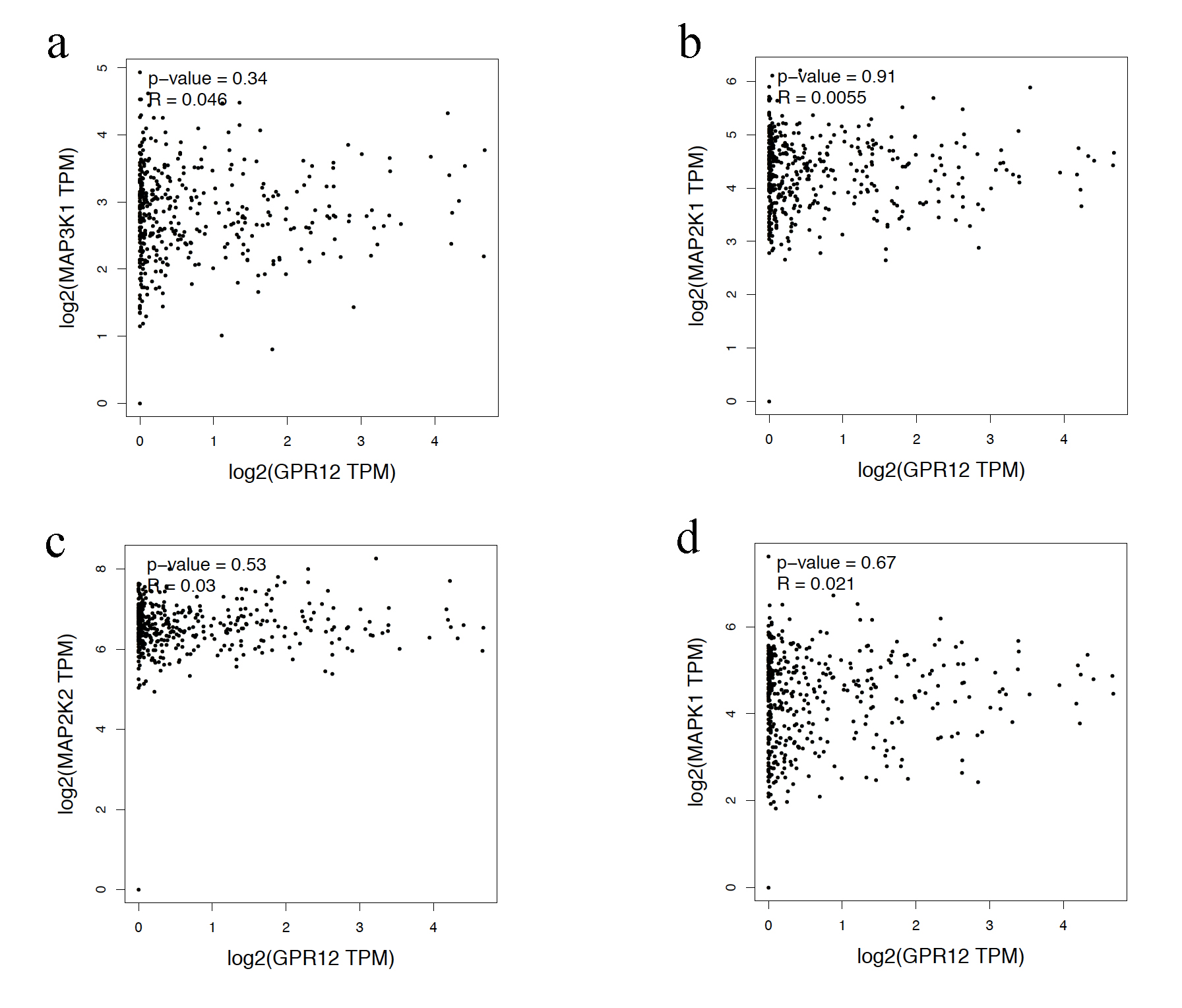

Supplement: Supplementary Figure 3 — Correlation analysis of GPR12 and ERK1/2 cascade in OV data from TCGA database. (A–D), Correlation analysis of GPR12 mRNA level with ERK1/2 cascade expression including MAP3K1 (A), MAP2K1 (B), MAP2K2 (C) and MAPK1 (D) by analyzing OV data from TCGA database. [file Image_3.jpeg]
