# Supplementary material for: GPR12 Inhibits Apoptosis in Epithelial Ovarian Cancer via the Activation of ERK1/2 Signaling
Source: Front Oncol. 2022 Jul 12;12:932689. doi: 10.3389/fonc.2022.932689 (PMC9316591; doi:10.3389/fonc.2022.932689)
Supplement: Supplementary Table 1 — Primers used in the reactions for clone PCR. [file Table_1.docx]

**Supplementary Table 1, A list of primers used in the reactions for clone PCR**

| Gene | Sequence (5` – 3`) |
| --- | --- |
| shGPR12-1#  -Forward | CCGGCTATTCCTTGATAGCGGATTACTCGAGTAATCCGCTATCAAGGAATAGTTTTTTG |
| shGPR12-1#  -Reverse | AATTCAAAAACTATTCCTTGATAGCGGATTACTCGAGTAATCCGCTATCAAGGAATAGT |
| shGPR12-2#  -Forward | CCGGCCAGATCTGTAAGATTGTGATCTCGAGATCACAATCTTACAGATCTGGTTTTTTG |
| shGPR12-2#  -Reverse | AATTCAAAAACCAGATCTGTAAGATTGTGATCTCGAGATCACAATCTTACAGATCTGGT |
| GPR12-Forward | GAATTGATCCTTCGAACTAGTATGAATGAAGACCTGAAGGTCAATTTAAG |
| GPR12-Reverse | TCGAGCTCAAGCTTCGAATTCCTACACATCACTGGGCGAGC |
